# Long-term results of carbidopa/levodopa enteral suspension across the day in advanced Parkinson’s disease: Post-hoc analyses from a large 54-week trial

**DOI:** 10.1016/j.prdoa.2022.100181

**Published:** 2022-12-20

**Authors:** Rajesh Pahwa, Jason Aldred, Aristide Merola, Niodita Gupta, Emi Terasawa, Viviana Garcia-Horton, David R. Steffen, Prasanna L. Kandukuri, Yanjun Bao, Omar Ladhani, Connie H. Yan, Vivek Chaudhari, Stuart H. Isaacson

**Affiliations:** a University of Kansas Medical Center, 3901 Rainbow Boulevard, Kansas City, KS 66160, USA; b Selkirk Neurology & Inland Northwest Research, PLLC, 610 S Sherman Suite 201, Spokane, WA 99202, USA; c Ohio State University, Wexner Medical Center, 480 Medical Center Drive, Columbus, OH 43210, USA; d AbbVie, Inc., 1 N. Waukegan Road, North Chicago, IL 60064, USA; e Analysis Group, Inc., 151 West 42nd Street, 23rd Floor, New York, NY 10036, USA; f University of Illinois at Chicago, 833 S. Wood Street, Chicago, IL 60612, USA; g Parkinson’s Disease and Movement Disorders Center, 951 NW 13th St, Boca Raton, FL 33486, USA

**Keywords:** Carbidopa/levodopa enteral suspension, Carbidopa/levodopa intestinal gel, Dyskinesia, Parkinson’s disease, Duopa, Duodopa, Long-term effectiveness, ADL, Activities of daily living, APD, Advanced Parkinson's disease, CGI-S, Clinical Global Impression of disease severity, CLES, Carbidopa/levodopa enteral suspension, HR, Hazard ratio, IRR, Incidence rate ratio, OFF, “Off” time, ON, “On” time, ON-woTD, “On” time without troublesome dyskinesia, ON-wTD, “On” time with troublesome dyskinesia, OR, Odds ratio, PD, Parkinson’s disease, PEG-J, Percutaneous endoscopic gastrojejunostomy, QoL, Quality of life, RCT, Randomized controlled trial, SD, Standard deviation

## Abstract

**Introduction:**

Carbidopa/levodopa enteral suspension (CLES) previously demonstrated reduction in total daily OFF from baseline by over 4 hours in advanced Parkinson’s disease patients across 54 weeks. Evidence on CLES’s long-term effectiveness on patterns of motor-symptom control throughout the day remains limited.

**Methods:**

We present post-hoc analyses of a large, open-label study of CLES monotherapy (N = 289). Diary data recorded patients’ motor states at 30-minute intervals over 3 days at baseline and weeks 4, 12, 24, 36, and 54. Adjusted generalized linear mixed models assessed changes from baseline at each timepoint for four outcome measures: time to ON without troublesome dyskinesia (ON-woTD) after waking, motor-symptom control as measured by motor states’ durations throughout the day, number of motor-state transitions, and presence of extreme fluctuations (OFF to ON with TD).

**Results:**

Patients demonstrated short-term (wk4) and sustained (wk54) improvement in all outcomes compared to baseline. At weeks 4 and 54, patients were more likely to reach ON-woTD over the course of their day (HR: 1.86 and 2.51, both P < 0.0001). Across 4-hour intervals throughout the day, patients also experienced increases in ON-woTD (wk4: 58–65 min; wk54: 60–78 min; all P < 0.0001) and reductions in OFF (wk4: 50–61 min; wk54: 56–68 min; all P < 0.0001). At weeks 4 and 54, patients’ motor-state transitions were reduced by about half (IRR: 0.53 and 0.49, both P < 0.0001), and fewer patients experienced extreme fluctuations (OR: 0.22 and 0.15, both P < 0.0001).

**Conclusion:**

CLES monotherapy was associated with significant long-term reductions in motor-state fluctuations, faster time to ON-woTD upon awakening, and increased symptom control throughout the day.

## Highlights

• In APD, fluctuations between patients’ motor states develop as disease progresses.
• Evidence on long-term impact of CLES on motor fluctuations across the day is limited.
• CLES was associated with fewer motor fluctuations and increased symptom control.
• Patients taking CLES had faster onset to good ON time after waking.
• CLES may improve patients’ quality of life by reducing unpredictable OFF periods.

## 1 Introduction

In advanced Parkinson’s disease (APD), response to treatment becomes less reliable and motor fluctuations develop as the disease progresses due to the narrowing therapeutic window of orally-administered levodopa, the gold standard treatment in Parkinson’s disease (PD) treatment [1], [2]. As patients’ disease advances, these motor fluctuations can impair patients’ ability to perform activities of daily living (ADL) and worsen their quality of life (QoL) [3], [4], [5], [6], [7], [8]. The number of motor-state transitions and extreme fluctuations experienced by patients can impact the predictability and consistency of their days and have important implications for QoL [4], [9], [10]. Additionally, the stability of these outcomes over time can have important implications for sustained treatment effects and improve patients’ ability to perform ADL and QoL.

Carbidopa/levodopa enteral suspension (CLES) is administered continuously via a percutaneous endoscopic gastrojejunostomy (PEG-J) tube that is connected to a portable pump. The pump delivers CLES into the jejunum, where levodopa absorption primarily occurs, bypassing gastric dysmotility and avoiding issues with erratic gastric emptying common in patients with APD [11], [12], [13], [14]. Through continuous and direct infusion into the jejunum, CLES enables steadier blood plasma levels and less variable absorption of levodopa compared to orals, potentially providing more continuous dopaminergic stimulation [15], [16], [17], [18]. Long-term effects of CLES on reduction of “off” time (OFF) and increase in “on” time without troublesome dyskinesia [ON-woTD; “Good ON”] have been documented in prior studies [19]. A large, prospective, 54-week, open-label study of CLES found that CLES monotherapy decreased mean total daily OFF by 4.4 hours (corresponding to a 65.6% decrease from baseline; P < 0.001), increased mean total daily ON-woTD by 4.8 hours (corresponding to a 62.9% increase from baseline; P < 0.001), and decreased mean total daily “on” time with troublesome dyskinesia (ON-wTD) by 0.4 hours (corresponding to a 22.5% decrease from baseline; P = 0.023).

Prior studies have also found large and significant short-term effects of CLES on several key outcomes including motor-state fluctuations, speed of onset of ON-woTD, and consistency of motor-symptom control as measured by motor-state duration throughout the day [20]. The aim of this study was to assess the long-term effectiveness of CLES monotherapy on speed of ON-woTD onset, motor-symptom control throughout the day, motor-state transitions, and extreme fluctuations from OFF to ON-wTD using data from a large, prospective, 54-week, open-label trial of CLES.

## 2 Methods

### 2.1 Study design and participants

This post-hoc analysis used data from the largest, prospective, open-label, phase III trial of CLES in patients with APD experiencing motor fluctuations despite optimized medical therapy [19]. The trial was conducted at 86 centers in 16 countries between January 2008 and June 2012. Eligible participants were age 30 or older, levodopa responsive, met UK Parkinson’s Disease Society Brain Bank diagnostic criteria, and experienced severe motor fluctuations defined as 3 hours of daily OFF at baseline (confirmed by the PD symptom diary), despite optimized treatment with available oral PD medications [19].

As previously described [19], the study consisted of a screening period followed by baseline assessments, a nasojejunal titration period, a PEG-J titration period, and a 54-week treatment period. A morning dose was followed by continuous infusion for approximately 16 hours, with additional rescue doses during the day if clinically indicated.

### 2.2 Patient diary assessments

The present analysis included all patients with evaluable 24-hour PD home-diary assessment of motor status. The diary data captured each patient’s motor states in 30-minute increments, over a 3-day period prior to each clinical visit, at baseline and weeks 4, 12, 24, 36, and 54. Patients were permitted to enter five possible motor states in their PD diary entries: OFF, ON without dyskinesia, ON with non-troublesome dyskinesia, ON-wTD, and asleep. As in prior studies, ON without dyskinesia and ON with non-troublesome dyskinesia were merged and renamed ON-woTD [21], [22].

For each patient diary day, the waking day was defined as a 16-hour period starting at wake-up time. Wake-up time was defined for each diary day as the start of the patient’s first 2 hours reported with no sleep after 3 AM [20], [22]. The second and third days prior to each visit were used in the analyses in order to ensure wake-up time was calculable for all patient diary days.

### 2.3 Outcome measures

Four outcome measures were evaluated using the patient diary data. For each outcome, their change from baseline was assessed at five timepoints (weeks 4, 12, 24, 36, and 54). The first outcome was the average time to ON-woTD onset after waking. The second outcome evaluated motor-symptom control, as measured by the mean durations of ON-woTD and OFF for each of the 4-hour intervals during their waking days: from waking to 4 hours, from 4 to 8 hours, from 8 to 12 hours, and from 12 to 16 hours after waking. The third outcome was the average number of motor-state transitions (transitions between OFF, ON-wTD, and ON-woTD) across the waking days. This outcome was also categorized into the following 5 groups: 0 to 3 transitions, 4 to 6 transitions, 7 to 9 transitions, 10 to 12 transitions, and >12 transitions. The fourth outcome was percentage of patients with the presence of extreme fluctuations (i.e., fluctuating between OFF and ON-wTD).

### 2.4 Statistical analyses

Adjusted generalized linear mixed models were fit with the respective outcome as the dependent variable, with subject-level random effects and fixed effects for each of the timepoints. The models were adjusted for baseline country of residence (U.S. vs non-U.S.), age, sex, and Clinical Global Impression of disease severity (CGI-S). Model estimates are reported for each timepoint compared to baseline.

The functional forms of the models were chosen according to the outcomes. Time to ON-woTD onset was modeled using a Cox proportional hazards model. Hazard ratios (HRs) comparing each timepoint to baseline were reported. The proportional hazards assumption was met for each timepoint. The motor-symptom control outcomes for duration of motor states throughout the day were modeled using linear regression models, with model estimates presented for the change from baseline in each motor state during each 4-hour interval. The number of motor-state transitions was modeled using a Poisson regression model, with incidence rate ratios (IRRs) presented. The presence of extreme fluctuations was modeled using a logistic regression model with binary distribution, with odds ratios (ORs) presented.

Descriptive statistics for the outcomes were calculated at baseline among all patients with evaluable diary data for at least one post-baseline timepoint, and separately at weeks 4, 12, and 54 among patients with evaluable diary data for the specified timepoint. At each timepoint, the descriptive statistics were compared to the corresponding baseline values for that sample of patients.

In addition to assessing the change from baseline in the outcomes described above, pairwise comparisons were conducted of the estimated change-from-baseline effects at each follow-up timepoint to assess the stability of the outcomes’ observed changes over a period of 54 weeks. For time to ON-woTD onset after waking, multiplicity of testing was adjusted using Tukey's all-pair comparisons [23]. For motor-state duration, number of motor-state transitions, and presence of extreme fluctuations, multiplicity of testing was adjusted using the Benjamini-Hochberg procedure [24].

## 3 Results

### 3.1 Study participants and baseline characteristics

Complete diary data at baseline and at least one post-baseline timepoint were available for 289 patients out of 321 in the study’s full analysis sample. Among the patients, 57.8% were male, and at baseline, patients had a mean ± standard deviation (SD) age of 63.9 ± 9.0 years, PD duration of 12.3 ± 5.6 years, daily dose of levodopa of 1095.4 ± 586.4 mg, and total daily OFF of 6.75 ± 2.39 hours (Table e1). Descriptive statistics for the outcomes were calculated at each timepoint among patients with evaluable diary data for the specified timepoint; specifically, this consisted of 251 patients with data at week 4, 261 patients with data at week 12, and 225 patients with data at week 54, respectively.

### 3.2 Time to ON-woTD onset after waking

**Fig. 1:**
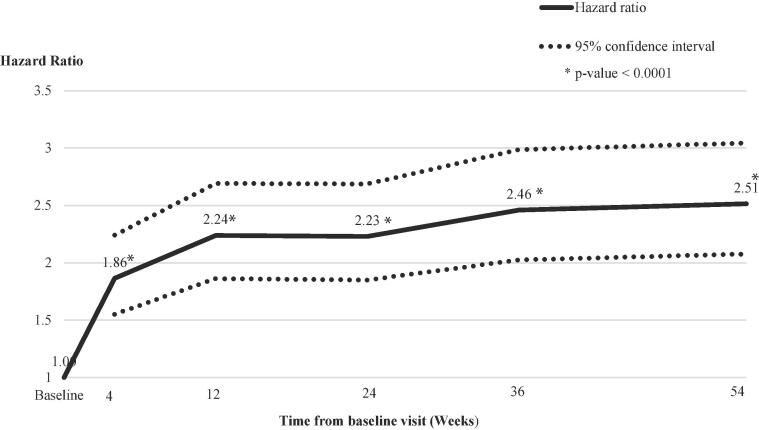
ON-woTD onset timing after waking: Hazard ratios compared to baseline over 54 weeks.

Patients were 1.86 times more likely to achieve ON-woTD after waking over the course of their day at week 4 compared to baseline (P < 0.0001). At weeks 12, 24, 36, and 54, patients were 2.23–2.51 times more likely to reach ON-woTD after waking than at baseline (Fig. 1; all P < 0.0001). Average time to ON-woTD onset after waking was 108 minutes at baseline, 65 minutes at week 4, 53 minutes at week 12, and 51 minutes at week 54. Pairwise comparisons of the estimated change-from-baseline effects at each follow-up timepoint supported the stability of this outcome over time (Table e2).

### 3.3 Motor-state duration

**Fig. 2:**
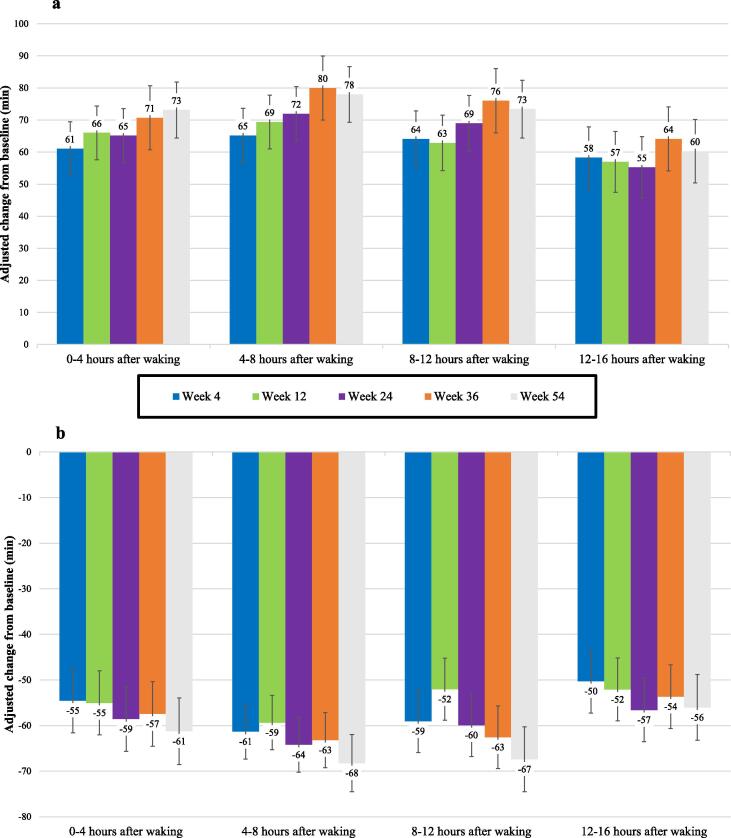
(a) Average change in duration of ON-woTD for selected 4-hour time intervals. (b) Average change in duration of OFF for selected 4-hour time intervals.

The mean duration in ON-woTD across the 4-hour intervals throughout the day increased from a range of 93–119 minutes at baseline (i.e., evaluated across the individual intervals) to a range of 152–183 minutes at week 4, to a range of 151–188 minutes at week 12, and to a range of 157–197 minutes at week 54. Mean duration in OFF decreased across the intervals from a range of 85–106 minutes at baseline to a range of 24–50 minutes at week 4, to a range of 27–50 minutes at week 12, and to a range of 17–44 minutes at week 54. Across the 4-hour intervals, increases in ON-woTD ranged from 55–80 minutes (Fig. 2a, all P < 0.0001), and decreases in OFF ranged from 50–68 minutes (Fig. 2b, all P < 0.0001) across all follow-up timepoints relative to baseline. Pairwise comparisons of the estimated change-from-baseline effects at each follow-up timepoint supported the stability of these outcomes over time (for each outcome, 95% of the p-values for the pairwise comparisons were non-significant, Table e2).

### 3.4 Number of motor-state transitions

There were approximately half as many motor-state transitions at the follow-up timepoints relative to baseline (IRR: 0.46–0.53, Fig. 3a, all P < 0.0001). The mean number of motor-state transitions was 8.13 at baseline and these decreased to 4.37 at week 4, 4.15 at week 12 and to 3.99 transitions at week 54. After 12 weeks of treatment, the percentage of patients with 0 to 6 transitions per day on average increased from less than 30% of patients at baseline to more than 75% of patients at week 12, and similar trends were observed between week 54 versus baseline (Fig. 3b). Pairwise comparisons of the estimated change-from-baseline effects at each follow-up timepoint supported the stability of this outcome over time (Table e2).

**Fig. 3a:**
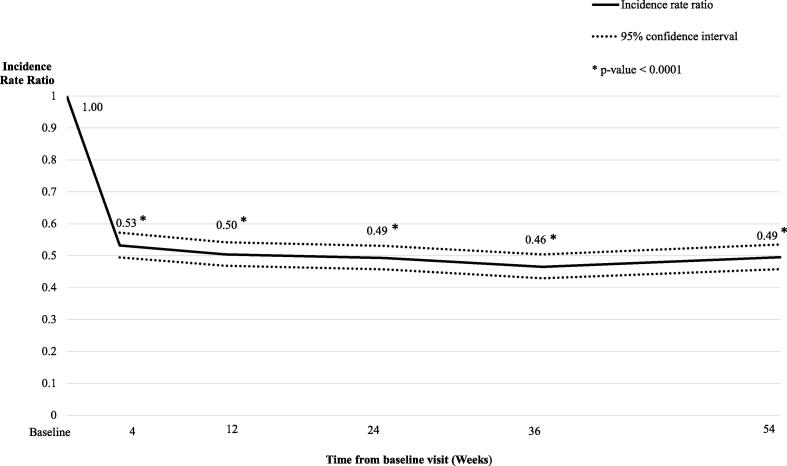
Motor-state transitions: Incidence rate ratios compared to baseline over 54 weeks.

**Fig. 3b:**
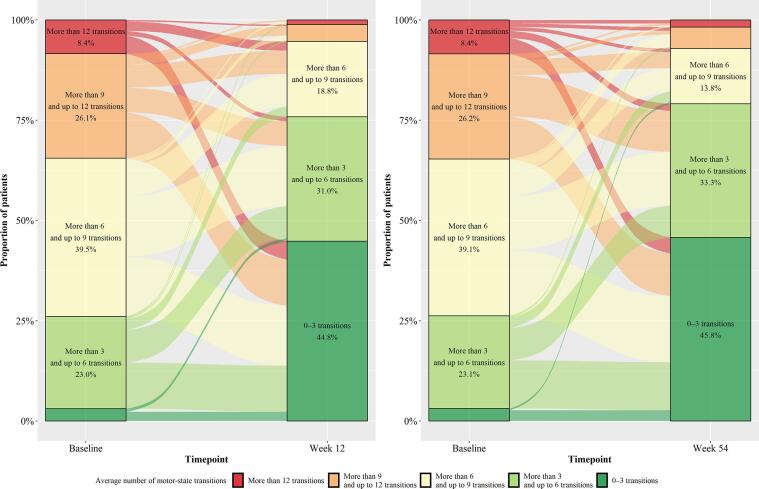
Average number of transitions between motor states during the waking day at baseline vs weeks 12 and 54.

### 3.5 Presence of extreme fluctuations

**Fig. 4:**
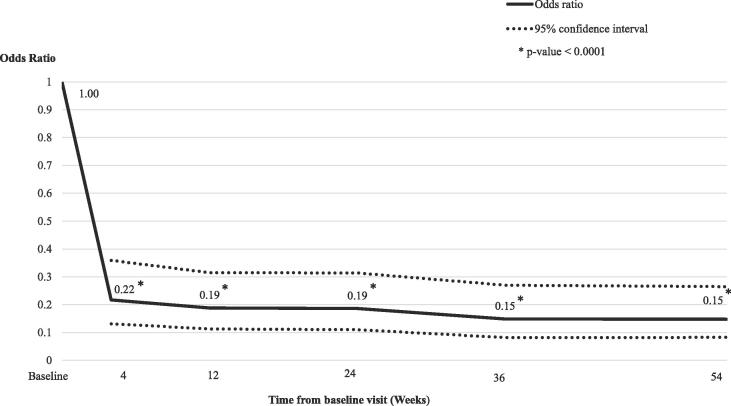
Presence of extreme fluctuations: Odds ratios compared to baseline over 54 weeks.

Across all follow-up timepoints, significantly fewer patients experienced extreme fluctuations relative to baseline (OR: 0.15–0.22, Fig. 4, all P < 0.0001). The percentage of patients who experienced an extreme fluctuation reduced from 31.8% at baseline to 10.8% of patients at week 4 (P < 0.0001), to 9.2% of patients at week 12 (P < 0.0001), and to 7.6% of patients at week 54 (P < 0.0001). Pairwise comparisons of the estimated change-from-baseline effects at each follow-up timepoint supported the stability of this outcome over time (all p-values were non-significant, Table e2).

## 4 Discussion

Patients demonstrated both short-term (4 weeks) and sustained (54 weeks) improvement in outcomes including time to ON-woTD onset upon awakening, consistency of motor-symptom control (i.e., motor-state duration), presence of extreme fluctuations, and fewer motor-state transitions throughout the waking day. These improvements in outcomes provide additional context to the longer-term impacts of CLES and supplement prior studies that demonstrated long-term benefits of CLES in real-world populations [25], [26], [27].

Patients reached ON-woTD after waking significantly faster for all follow-up timepoints compared to baseline. This was seen even though patients were not using the CLES pump at nighttime and had mean CLES dose that remained relatively constant across timepoints [19]. A prior post-hoc analysis of a 12-week randomized controlled trial (RCT) of CLES reported that, after 12 weeks, three times as many CLES-treated patients achieved ON-woTD within 30 minutes of waking [20]. However, that prior post-hoc analysis of a shorter trial did not estimate the benefit across CLES-treated patients in terms of average time to ON-woTD onset or longer-term effects beyond 12 weeks. This analysis fills this gap by quantifying the large and significant effects across patients with time-to-event analyses and demonstrated the persistence of these benefits over time. The negative effects of morning akinesia on patient ADL and QoL have been repeatedly described in the literature [4], [5], [8]. Specifically, morning akinesia has been found to decrease QoL on several dimensions and to impair ADL such as rising from bed, dressing, bathing, toileting, preparing breakfast and starting their day, and constitutes a major burden on patients and caregivers. The more rapid onset of ON-woTD in the morning may reflect bypassing of the stomach and direct delivery to the jejunum, avoiding the delayed gastric emptying of oral doses due to gastroparesis. The benefit of non-oral routes of medication in avoiding delayed gastric absorption was also seen in a prior study that found that patients receiving subcutaneous apomorphine injection had faster time to ON following morning apomorphine injection compared to oral levodopa [28]. The administration of CLES directly into the jejunum, where levodopa absorption primarily occurs, therefore results in faster absorption of levodopa, which may reduce the time in morning OFF and achieve quicker onset of morning ON-woTD [29]. By allowing patients to reach ON-woTD significantly faster in the morning and maintaining this benefit over longer duration, CLES has the potential to increase patients’ independence in ADL, reduce caregiver burden, and improve patients’ QoL.

For motor-symptom control, patients experienced large increases in ON-woTD and large reductions in OFF consistently throughout the waking day. The aforementioned 12-week RCT analysis found increases in ON-woTD of between 33 and 58 minutes and reductions in OFF of between 34 and 52 minutes across the four 4-hour periods throughout the day in CLES-treated patients from baseline to week 12 [20]. This post-hoc analysis found numerically larger increases in ON-woTD of between 55 and 80 minutes and decreases in OFF of between 50 and 68 minutes consistently across the four 4-hour periods throughout the day across all follow-up timepoints from week 4 to week 54 relative to baseline, and shows that these benefits persist in the long term.

The number of motor-state transitions experienced after treatment initiation was reduced by about half across the follow-up timepoints. Motor-state transitions constitute an important burden for patients and their caregivers due to the unpredictability during the patient’s day. For instance, frequent transitions prevent patients from anticipating if they will be able to perform an activity, go out, or make an appointment at a specific time, making their daily life less predictable. Unexpected OFF episodes and variability in ON-woTD can make planning daily activities difficult for patients and their caregivers [7]. Therefore, treatments that provide consistent motor-state stability or minimize daily motor-state transitions may have important implications for patients’ and caregivers’ QoL.

Finally, across all follow-up timepoints, significantly fewer patients experienced extreme fluctuations (i.e., between OFF and ON-wTD) relative to baseline. Our findings that this benefit is sustained over 54 weeks is clinically notable given the damaging effects of extreme fluctuations on patients’ QoL, as dyskinesias, including those before OFF, may lead to exhaustion, depression, limitations in social life, and reduced QoL across several dimensions; dyskinesias may additionally pose risk of injury for patients and caregivers, and increases in the burden of care and healthcare costs [4], [9], [10]. This finding is also meaningful given the natural progression of the disease and its expected evolution towards more extreme fluctuations as PD advances [30].

The long-term effectiveness of CLES on several key temporal motor-symptom outcomes with potential implications for patient ADL and QoL, was examined among a large sample of patients with APD. Detailed analysis of fluctuations and temporal patterns in motor states throughout the day was possible due to the granular nature of the patient PD diary assessments. However, this analysis is subject to some limitations. First, the generalizability of the results may be limited. The clinical trial data used in this analysis required that patients met specific inclusion and exclusion criteria. As a result, these patients may not be representative of patients with APD in the real world. It should be noted, however, that subjects in this trial had similar characteristics of patients with APD. Second, the diary data are patient-reported. The patient-reported nature of these outcomes may create variation in motor-state reporting within and across individual patients. Third, the clinical trial analyzed here is an open-label trial with no control group. Fourth, this was a post-hoc analysis and therefore may be subject to limitations of such analyses including multiplicity and spurious associations [31].

Our longitudinal analyses of daily patterns support the long-term effectiveness of CLES in increasing time to ON-woTD onset after waking, improving motor-symptom control as measured by motor-states’ duration throughout the day, and reducing motor-state transitions and extreme fluctuations. The findings suggest that CLES, which provides continuous dopaminergic stimulation over 16 hours of patients’ waking days, improves the predictability of motor states across the waking day in patients’ daily lives and provides consistent motor-symptom control over the long term, which has important implications for improving patients’ QoL and ability to perform ADL. Future research should evaluate the effectiveness of CLES on these outcomes among a real-world population.

## CRediT authorship contribution statement

**Rajesh Pahwa:** Conceptualization, Methodology, Writing – original draft, Writing – review & editing. **Jason Aldred:** Conceptualization, Methodology, Writing – original draft, Writing – review & editing. **Aristide Merola:** Conceptualization, Methodology, Writing – original draft, Writing – review & editing. **Niodita Gupta:** Conceptualization, Methodology, Resources, Writing – original draft, Writing – review & editing, Supervision, Project administration, Funding acquisition. **Emi Terasawa:** Conceptualization, Methodology, Software, Validation, Formal analysis, Investigation, Data curation, Writing – original draft, Writing – review & editing, Visualization. **Viviana Garcia-Horton:** Conceptualization, Methodology, Software, Validation, Formal analysis, Investigation, Data curation, Writing – original draft, Writing – review & editing, Visualization. **David R. Steffen:** Conceptualization, Methodology, Software, Validation, Formal analysis, Investigation, Data curation, Writing – original draft, Writing – review & editing, Visualization. **Prasanna L. Kandukuri:** Conceptualization, Methodology, Resources, Writing – original draft, Writing – review & editing, Supervision, Project administration, Funding acquisition. **Yanjun Bao:** Conceptualization, Methodology, Resources, Writing – original draft, Writing – review & editing, Supervision, Project administration, Funding acquisition. **Omar Ladhani:** Conceptualization, Methodology, Resources, Writing – original draft, Writing – review & editing, Supervision, Project administration, Funding acquisition. **Connie H. Yan:** Conceptualization, Methodology, Resources, Writing – original draft, Writing – review & editing, Supervision, Project administration, Funding acquisition. **Vivek Chaudhari:** Conceptualization, Methodology, Resources, Writing – original draft, Writing – review & editing, Supervision, Project administration, Funding acquisition. **Stuart H. Isaacson:** Conceptualization, Methodology, Writing – original draft, Writing – review & editing.

## Declaration of Competing Interest

The authors declare the following relationships, which may be considered as potential competing interests: Rajesh Pahwa has received consulting fees from AbbVie, Acadia, Acorda, Adamas, Cynapses, Global Kinetics, Lundbeck, Neurocrine, Pfizer, Sage, Sunovion, Teva Neuroscience, and US World Meds. He has received research grants from AbbVie, Adamas, Avid, Biotie, Boston Scientific, Civitas, Cynapses, Kyowa, National Parkinson Foundation, NIH/NINDS, and Parkinson Study Group. Jason Aldred has been a consultant and received honorarium from AbbVie, Acorda, Adamas, Allergan, Boston Scientific, Teva, Medtronic, and US World Meds. He has also received research support from NINDS, Abbott, AbbVie, Acadia, Biogen, Boston Scientific, Denali, Impax/Amneal, Sunovion, Neuroderm, Novartis, and Theravance. Aristide Merola has received support from the NIH (KL2 TR001426) and speaker or consultancy honoraria from CSL Behring, AbbVie, Abbott, and Cynapsus Therapeutics. He has received grant support from Lundbeck and AbbVie. Prasanna L. Kandukuri, Yanjun Bao, Omar Ladhani, and Connie H. Yan are employees of AbbVie and may own stocks/shares in the company. Niodita R. Gupta is a former employee of AbbVie, currently employed by Johnson and Johnson, and may hold AbbVie stock. Vivek S. Chaudhari is a former employee of AbbVie, currently employed by EMD Serono, Inc. and may hold AbbVie stock. Emi Terasawa, Viviana Garcia-Horton, and David R. Steffen are employees of Analysis Group, Inc., which has received consultancy fees from AbbVie. Stuart H. Isaacson has received honoraria for CME, consultant, research grants, and/or promotional speaker on behalf of: AbbVie, Acadia, Acorda, Adamas, Addex, Allergan, Amarantus, Axovant, Benevolent, Biogen, Britannia, Cerecor, Eli Lilly, Enterin, GE Healthcare, Global Kinetics, Impax, Intec Pharma, Ipsen, Jazz, Kyowa, Lundbeck, Michael J. Fox Foundation, Neurocrine, Neuroderm, Parkinson’s Study Group, Pharma2B, Roche, Sanofi, Sunovion, Teva, Theravance, UCB, US World Meds, and Zambon.

## Appendix A Supplementary data

Supplementary Tables e1 and e2 Supplementary Table e1. Baseline characteristics. Supplementary Table e2. P-values of pairwise comparisons of the estimated effects at each follow-up timepoint (adjusted models).
